# A qualitative evaluation of factors influencing Tumor Treating fields (TTFields) therapy decision making among brain tumor patients and physicians

**DOI:** 10.1186/s12885-024-12042-x

**Published:** 2024-04-25

**Authors:** Priya Kumthekar, Madison Lyleroehr, Leilani Lacson, Rimas V. Lukas, Karan Dixit, Roger Stupp, Timothy Kruser, Jeff Raizer, Alexander Hou, Sean Sachdev, Margaret Schwartz, Jessica Bajas PA, Ray Lezon, Karyn Schmidt, Christina Amidei, Karen Kaiser

**Affiliations:** 1 Department of Neurology, Northwestern University Feinberg School of Medicine, Abbott Hall Suite 1122 710 N Lake Shore Drive, Chicago, IL 60611 USA; 2 Lou and Jean Malnati Brain Tumor Institute, Robert H. Lurie Comprehensive Cancer Center of Northwestern University, Chicago, United States; 3 Department of Neurological Surgery, Northwestern University Feinberg School of Medicine, 676 N. St. Clair Street, Suite 2210, Chicago, IL 60611 USA; 4 Department of Radiation Oncology, Northwestern University Feinberg School of Medicine, 676 N. St. Clair Street, Suite 1820, Chicago, IL 60611 USA; 5 Northwestern University Feinberg School of Medicine, 420 E. Superior St, Chicago, IL 60611 USA; 6 Department of Human Oncology, University of Wisconsin Carbone Cancer Center, 600 Highland Ave, Madison, WI 53705 USA; 7 Equal Hope, 300 South Ashland Avenue, Chicago, IL 60607 USA

**Keywords:** Glioblastoma, Tumor treating fields, Decision-making

## Abstract

**Background:**

Tumor Treating Fields (TTFields) Therapy is an FDA-approved therapy in the first line and recurrent setting for glioblastoma. Despite Phase 3 evidence showing improved survival with TTFields, it is not uniformly utilized. We aimed to examine patient and clinician views of TTFields and factors shaping utilization of TTFields through a unique research partnership with medical neuro oncology and medical social sciences.

**Methods:**

Adult glioblastoma patients who were offered TTFields at a tertiary care academic hospital were invited to participate in a semi-structured interview about their decision to use or not use TTFields. Clinicians who prescribe TTFields were invited to participate in a semi-structured interview about TTFields.

**Results:**

Interviews were completed with 40 patients with a mean age of 53 years; 92.5% were white and 60% were male. Participants who decided against TTFields stated that head shaving, appearing sick, and inconvenience of wearing/carrying the device most influenced their decision. The most influential factors for use of TTFields were the efficacy of the device and their clinician’s opinion. Clinicians (*N* = 9) stated that TTFields was a good option for glioblastoma patients, but some noted that their patients should consider the burdens and benefits of TTFields as it may not be the desired choice for all patients.

**Conclusions:**

This is the first study to examine patient decision making for TTFields. Findings suggest that clinician support and efficacy data are among the key decision-making factors. Properly understanding the path to patients’ decision making is crucial in optimizing the use of TTFields and other therapeutic decisions for glioblastoma patients.

## Introduction

Glioblastoma is the most common malignant primary brain tumor in adults, accounting for over 50% of all gliomas, with an annual incidence rate of 3 to 4 cases per 100,000 people, resulting in 240,000 newly diagnosed cases worldwide each year [1, 2]. Since 2005, the standard treatment for glioblastoma has been surgical resection followed by radiotherapy with concurrent and adjuvant temozolomide (TMZ). For newly diagnosed glioblastomas, the addition of TMZ to surgery and radiation therapy prolongs median survival from 12.1 to 14.6 months and increases the five-year survival rate from 2 to 10% [3].

In addition to the current standard of care, patients with glioblastomas can receive a more recently approved therapy called Tumor Treating Fields Therapy (TTFields) (Optune™). TTFields utilizes a portable, battery-operated device that delivers a low-intensity, alternating electric field to the tumor via surface electrodes (transducer arrays) [4]. The electric field disrupts the normal mitotic process for tumor cells and leads to cancer cell death [5]. In patients with recurrent glioblastoma brain tumors, TTFields has shown clinical efficacy comparable to that of active chemotherapies, without many of the side effects of chemotherapy [6, 7]. TTFields was first FDA approved in the recurrent setting after it showed non-inferiority in a phase 3 trial compared to “physician’s best choice” treatment [8]. Subsequently, a first line randomized Phase 3 trial compared TTFields plus TMZ to TMZ alone among 695 glioblastoma patients who had completed radiochemotherapy. The TTFields treatment arm showed a significant improvement in both progression-free survival (median progression-free survival was 6.7 months in the TTFields plus TMZ group and 4.0 months in the TMZ alone group) and overall survival (median overall survival 20.9 months versus 16.0 months), leading to FDA approval of TTFields in the first line setting [9, 10]. Side effects reported in the TTFields plus TMZ group were limited to mild to moderate skin toxicity under the transducer arrays [10]. Following FDA approval to use TTFields for glioblastoma and, subsequently, mesothelioma [11], the National Comprehensive Cancer Network (NCCN) added TTFields to its guidelines for evidence-based management of new or recurrent glioblastoma in 2018 [12, 13].

Despite regulatory and national guideline support for TTFields, evidence demonstrating improved survival with TTFields, no severe side effects from the device, and limited treatments for glioblastoma, not all patients who qualify for TTFields choose to use the device. Moreover, there has been reluctance among neuro-oncologists to adopt TTFields [12]. This qualitative study aimed to grow our understanding of glioblastoma patients’ decision on whether or not to use TTFields, the role of clinicians in their decisions, and clinicians’ views of TTFields.

## Materials and methods

### Patient enrollment

The study protocol was approved by the institutional review board of Northwestern University and all patients enrolled on study provided written informed consent prior to any study-related procedures. Patients with a glioblastoma receiving treatment at the Malnati Brain Tumor Institute at the Lurie Comprehensive Cancer Center of Northwestern University who had been offered treatment with TTFields and had decided whether to initiate TTFields therapy were eligible to participate in the study. Other eligibility criteria included: able to speak and understand English, age 18 or older, and cognitively able to complete a study interview. We aimed to interview up to 50 glioblastoma patients who had been offered TTFields. In 2020, 180 new glioblastoma patients were seen at the Malnati Brain Tumor Institute and 51 TTFields prescriptions were written.

Patients were enrolled consecutively between June of 2020 and February of 2021, the entirety of which occurred during Covid-19 pandemic; therefore, consenting and interviews occurred remotely. In the first month of the project, clinicians provided the names of glioblastoma patients who had been offered TTFields for study inclusion; subsequently, a study coordinator (co-author LL) identified glioblastoma patients in the electronic medical record who had been offered or who had decided to use TTFields. Clinicians then confirmed patients were physically and cognitively able to participant and the study coordinator contacted eligible patients via telephone, explained the study, and obtained informed consent electronically. The approximately 30-minute study interview was subsequently conducted via phone by the study coordinator, who had extensive experience conducting patient interviews. A semi-structured interview guide was used to gather patient input on their views of TTFields, factors that influenced their TTFields decision, and their perceptions of whether their clinician wanted them to use TTFields. Patients also completed the Control Preference Scale, which is a one-item measure of preferred role in medical decision making that has been tested and validated with cancer patients [14–16]. Patients received a $25 Visa gift card following participation. Interviews were audio recorded to ensure comprehensive capture of all relevant patient-reported information.

### Clinician sample

Clinicians in the Malnati Brain Tumor Institute of Northwestern University who offer and prescribe TTFields therapy received an invitation via email to participate in a study interview. This included physicians (neuro-oncologists) and advanced practitioners (Advanced Practice Nurses and Physicians Assistants). All eligible physicians and advanced practitioners interviewed were specifically trained and certified to prescribe tumor treating fields by Novocure through the company-run training program. This is required to be able to prescribe this device to patients. Clinician participants provided verbal consent via telephone prior to participating in a telephone interview. As with the patient interviews, clinicians’ interviews were audio recorded. A semi-structured interview guide was used to gather their views of TTFields. Clinicians were not compensated for participating.

### Data analysis

The interviewer entered detailed notes into an Excel database following each interview. The database became the basis for a preliminary analysis of each interview question. Notes for each question were reviewed by the study coordinator, an experienced qualitative analyst (co-author ML), and a qualitative researcher (co-author KK) to identify preliminary themes. Next, the three study team members reviewed the interview transcripts to finalize themes, confirm themes were comprehensive and reflected the data, and to identify supporting quotations.

## Results

### Patient results

One-hundred and five glioblastoma patients were identified via physician recommendation or medical record review between June of 2020 and February of 2021. Of these, a total of 11 (10.5%) patients were deemed ineligible because they were not offered TTFields (*n* = 2), had gone to another center for treatment (*n* = 1), had entered hospice (*n* = 6), or died prior to recruitment (*n* = 2). Twenty-one patients (20.0%) were excluded per clinician recommendation. Reasons for clinician-recommended exclusion included cognitive issues, speech difficulties, and illness severity. Twenty (19.1%) patients were unresponsive to recruitment efforts, and 13 (12.4%) patients declined to participate. Individual interviews were conducted with 40 (38.1%) individuals with glioblastoma. Among the 40 participants (Table 1), the mean age was 53 years (range 21–77 years). Most participants were white (92.5%), married (80.0%), and male (60.0%). The study coordinator confirmed each patient’s TTFields status at the time of recruitment. Thirty-two participants (80.0%) chose to pursue TTFields therapy, while eight (20.0%) did not use TTFields. The TTFields group and the no-TTFields group were similar in age and gender. Patients who declined TTFields were less likely to use TTFields resources (Table 1); however, we would expect that TTFields users would be more apt to seek out information about TTFields.

**Table 1:** Patient Sample Characteristics (*N* = 40)

Characteristic	Total Sample *N* = 40 Number (%)	Utilized TTF *n* = 32 Number (%)	Declined TTF *N* = 8 Number (%)
Gender			
Male	24 (60.0)	19 (59.4)	5 (62.5)
Female	16 (40.0)	13 (40.6)	3 (37.5)
Age - Mean (Standard deviation)	53.3 (15.1)	53.4 (14.9)	52.9 (17.0)
Ethnicity			
Non-Hispanic	35 (87.5)	28 (87.5)	7 (87.5)
Hispanic	5 (12.50)	4 (12.5)	1 (12.5)
Race			
White	37 (92.5)	29 (90.6)	8 (100.0)
Asian	3 (7.5)	3 (9.4)	0 (0.0)
Marital Status			
Married	32 (80.0)	26 (81.3)	6 (75.0)
Never married	4 (10.0)	3 (9.4)	1 (12.5)
In a committed relationship	3 (7.5)	2 (6.3)	1 (12.5)
Widowed	1 (2.5)	1 (3.1)	0 (0.0)
Highest Year of Education			
High school/GED	3 (7.5)	2 (6.3)	1 (12.5)
Some college/Tech degree/AA	5 (12.5)	5 (15.6)	0 (0.0)
College	14 (35.0)	12 (37.5)	2 (25.0)
Advanced degree	18 (45.0)	13 (40.6)	5 (62.5)
Employment Status			
Full-time employed	12 (30.0%)	10 (31.3)	2 (25.0)
Part-time employed	3 (7.5%)	2 (6.3)	1 (12.5)
Unemployed	7 (17.5%)	5 (15.6)	2 (25.0)
Retired	12 (30.0%)	9 (28.1)	3 (37.5)
On disability	4 (10.0%)	4 (12.5)	0 (0.0)
On leave of absence	2 (5.0%)	2 (6.3)	0 (0.0)
Urban/Rural Status			
Suburban	29 (72.5)	25 (78.1)	6 (75.0)
Urban/Metropolitan	8 (20.0)	7 (21.9)	1 (12.5)
Rural	1 (2.5)	0 (0.0)	1 (12.5)
Current Living Situation^*a*^			
With partner With children With a caretaker With parents Alone	35 (87.5) 13 (32.5) 4 (10.0) 2 (5.0) 1 (2.5)	28 (87.5) 10 (31.3) 3 (9.4) 2 (6.3) 1 (3.1)	7 (87.5) 3 (37.5) 1 (12.5) 0 (0.0) 0 (0.0)
Current Activity Level			
Normal activity and no symptoms Some symptoms that do not require rest Requires rest < 50% of the day Requires rest > 50% of the day	14 (35.0) 10 (25.0) 12 (30.0) 4 (10.0)	11 (34.4) 7 (21.9) 10 (31.3) 4 (12.5)	3 (37.5) 3 (37.5) 2 (25.0) 0 (0.0)
Use of Resources^a^			
None Novocure Paper Brochure Education videos Novocure website Novocure Ambassador Program Acquainted with TTFields user In person support group(s) Novocure Buddy program Online/Facebook support group(s)	3 (7.5) 26 (65.0) 25 (62.5) 20 (50.0) 16 (40.0) 13 (32.5) 12 (30.0) 5 (12.5) 4 (10.0)	1 (3.1) 24 (75.0) 23 (71.9) 18 (56.3) 16 (50.0) 11 (34.4) 12 (37.5) 5 (15.6) 2 (6.3)	2 (25.0) 2 (25.0) 2 (25.0) 2 (25.0) 0 (0.0) 2 (25.0) 0 (0.0) 0 (0.0) 2 (25.0)

^*a*^Respondents could choose > 1

Control Preference Scale results are shown in Table 2. In both groups, patients most often preferred to make decisions for themselves after considering their doctors opinion (*n* = 15, 46.9% among patients who utilized TTFields, *n* = 5, 62.5% among patients who declined TTFields). Six (18.8%) of the patients who chose TTFields preferred to have their doctor make their medical decisions either with consideration for the patient’s opinion (*n* = 3, 9.4%) or on their own (*n* = 3, 9.4%). In contrast, none of the patients who declined TTFields preferred to have their doctor make their decisions.

**Table 2:** Desired Decision-Making Role (*N* = 40)

Characteristic	All (*N* = 40)	Utilized TTFields (*n* = 32)	Declined TTFields (*n* = 8)
Control Preferences Scale			
Patient makes decision Patient makes decision after considering doctor’s opinion Patient and doctor make decision Doctor makes decision but considers patient’s opinion Doctor makes decision	3 (7.5%) 20 (50.0%) 11 (27.5%) 3 (7.5%) 3 (7.5%)	2 (6.3%) 15 (46.9%) 9 (28.1%) 3 (9.4%) 3 (9.4%)	1 (12.5%) 5 (62.5%) 2 (25.0%) 0 (0.0%) 0 (0.0%)

### Participants who chose TTFields

Participants who chose TTFields noted downsides associated with the treatment. The most frequently mentioned downside of TTFields was the burden of changing the TTFields arrays, which happened frequently (every few days) and could be painful. The heat of the device on one’s head was also noted as a downside, along with the discomfort of wearing the cap and the burden on caregivers who assist them when changing their cap and arrays. Some users of TTFields experience skin reactions, such as blisters or itching. Participants also noted it could be difficult to sleep or shower with the device, and carrying the device was cumbersome.

However, many of the patients who chose TTFields came to accept the burdens of using the device. For example, some noted that shaving their head for TTFields was easy and/or worth the trouble. “The head shaving part was easy because I had lost most of my hair already.” Another participant said, “I used it (TTFields) for two years, and if they told me that I had to re-shave my head and use it longer, I would do it.” Likewise, participants who chose TTFields said that the appearance of the device could be bothersome, but they were not deterred by it. In fact, one participant had a positive experience due to the visible nature of TTFields: “Pretty much right after I was fitted for it, I think the next day, I was back at work. So, it was kind of awkward at first, but I actually had some good conversations with people about it in the days and weeks after that.”

For the patients who chose TTFields, the efficacy of TTFields was the most frequently cited reason for choosing to use the device. As shown in Table 3, they described efficacy in several ways, including extension of life, shrinking one’s tumor, and the device being supported by clinical studies. Extension of life was cited by over a third (*n* = 13, 40.6%) of those who chose TTFields (Table 3). One participant stated, “I don’t want to die. And I feel that if I don’t have this, that I will die.” Another participant spoke of the value of extending life even with potential negative effects: “Am I willing to be slightly inconvenienced for an additional five months of life? Yeah, I’ll go with that deal.” For others, clinical evidence supporting the efficacy of TTFields, particularly the extension of life, was pivotal in their decision to undergo TTFields. One participant stated that the biggest factor in their decision was “without question, FDA approved that this treatment does extend your life by a significant percentage.”

The second most influential factor reported by those who chose TTFields was their doctor’s opinion (*n* = 9, 28.1%). As one participant stated, “We (participant and their spouse) absolutely believe in both the physicians who recommended it (TTFields). So, we really trust them a lot.” All participants who reported that their doctor’s opinion was the most influential factor in their decision to undergo TTFields also reported that their doctor recommended TTFields.

Familial considerations were cited as the most influential factor for four (12.5%) participants. Three of the four people in this group noted that they wanted to extend time with their families. Several other influences were mentioned by one person each (Table 3). For example, one participant said she chose TTFields after hearing about the experience of a family friend who had used TTFields and recommended it despite the inconveniences of wearing the device.

**Table 3:** Most Influential Factors in Deciding to Use TTFields (*N* = 32)

Reason for using TTFields	Number (%) of participants who reported this reason^a^
Efficacy	23 (71.9%)
Extension of life	13 (40.6%)
Supported by clinical studies	4 (12.5%)
Shrink tumor	3 (9.4%)
Prevent recurrence	2 (6.3%)
Seemed like it would help	1 (3.1%)
Doctor’s opinion	9 (28.1%)
Family considerations	4 (12.5%)
Low risk/side effects	2 (6.3%)
Best/only option	2 (6.3%)
Having normal life	1 (3.1%)
Extended period of quality life	1 (3.1%)
Works with chemo	1 (3.1%)
Avoiding additional surgery	1 (3.1%)
Had questions answered	1 (3.1%)
Experience of friend	1 (3.1%)

^*a*^Respondents could report more than 1 factor

### Participants who declined TTFields

For those glioblastoma patients who chose not to use TTFields, the most influential factor in their decision was the requirement to shave their head (*n* = 4, 50.0%) (Table 4). “I know some people probably think that’s kind of silly, like, ‘You have cancer, so who cares if you have hair or not?’ But it’s just been really hard for me.” Another influential factor was the visibility of TTFields (*n* = 3, 37.5%). For example, two participants did not like the idea of wearing the device at work. One of them said, “I was still working, and at that time, I did not share with them everything that was going on. So, it would have definitely exposed things, and who knows what would have happened after that.”

**Table 4:** Most Influential Factors in Declining TTFields (*N* = 8)

Reason for declining TTFields	Number (%) of participants who reported this reason^a^
Shaving head	4 (50.0%)
Visibility of TTFields	3 (37.5%)
Inconvenience of wearing/carrying device	2 (25.0%)
Concern of interaction with other treatment	2 (25.0%)
Increased lifespan does not outweigh negative impact	1 (12.5%)
Doesn’t improve quality of life	1 (12.5%)
Didn’t think it was needed	1 (12.5%)
Had a lot going on	1 (12.5%)

^*a*^Respondents could choose > 1

Other reasons for declining TTFields included the inconvenience of continuously wearing the device and carrying the battery pack. One participant expressed a personal concern about the idea of simultaneously receiving chemo and TTFields, while another reported that multiple doctors recommended against going on TTFields because of an existing surgical implant. Additional reasons for not going on TTFields included thinking it would not be needed, that it would not improve quality of life, and that the increased lifespan would not outweigh the negative impact of TTFields. As one participant explained:

Yes, there’s no negative impact, I guess, physically. However, the idea of having something attached to my head and then having to carry around the battery pack doesn’t really mesh well with my relatively physical quality of life…(TTFields) would actually have quite significant negative impacts on how I live my life.

### Questions about TTFields

When asked if there was anything about TTFields that was confusing to them, most participants (*N* = 33, 85.5%) said no. The aspects of TTFields reported as unclear by seven (17.5%) participants are shown in Table 5. Participants questions centered around efficacy of the device, how the device works, and what to do if the device gets wet.

**Table 5:** What is Confusing about TTF? (*N* = 7)

What is confusing about TTF?
Total time wearing the device and time each day required to wear the device. “How long would I have to (wear it)? Was it like 24 months? Or something around that? On a daily basis, how long do you wear it? Do you ever take it off?” (Participant who chose NOT to use TTF)
Does efficacy vary by age of user? “If it were in some way shown that wearing the TTF at age 43 is going to extend my life by a year, or two years, or three years, relative to the average that was presented, which was about four months, then I might be more interested or willing to try wearing it.” (Participant who chose NOT to use TTF)
How do the rays hit the tumor when the tumor is small? (Participant who chose to use TTF)
How can you evaluate if it is working? (Participant who chose to use TTF)
How do you manage the device if it is hot outside or if it is raining? (Participant who chose to use TTF)
What should he do if he gets caught in the rain? What are the risks? (Participant who chose to use TTF)
How do pulses and currents work in his head to stop the cancer from dividing? Why does he get burns from the device? (Participant who chose to use TTF)

### Participants views of extension of life from TTFields

We asked participants to describe their understanding of how long TTFields would extend one’s life. Of the 30 participants who answered this question (the question was added to the interview guide after 9 interviews had been completed; one participant chose to skip this question), the most common response was that they did not know how long TTFields extends life (*n* = 12, 40.0%). The next most common response was that TTFields extended life several months (e.g., “2–4 months”, “3–6 months”) (*n* = 8, 26.6%). Five participants (16.6%) said that they did not have a specific number in mind, but they believed that TTFields extended life. Three participants (10.0%) said TTFields extend life by years (“2 years”, “2–5 years”, “3–14 years”) and two participants (6.6%) provided an answer based on a percentage (e.g., 20–30%, 15–18%); it was unclear what those percentages meant.

Six participants who did not choose TTFields answered the question about extension of life. Of these, 4 (66.6%) said they did not know the extension of life provided by TTFields.

### Impact of physician recommendations for TTFields

**Fig. 1:**
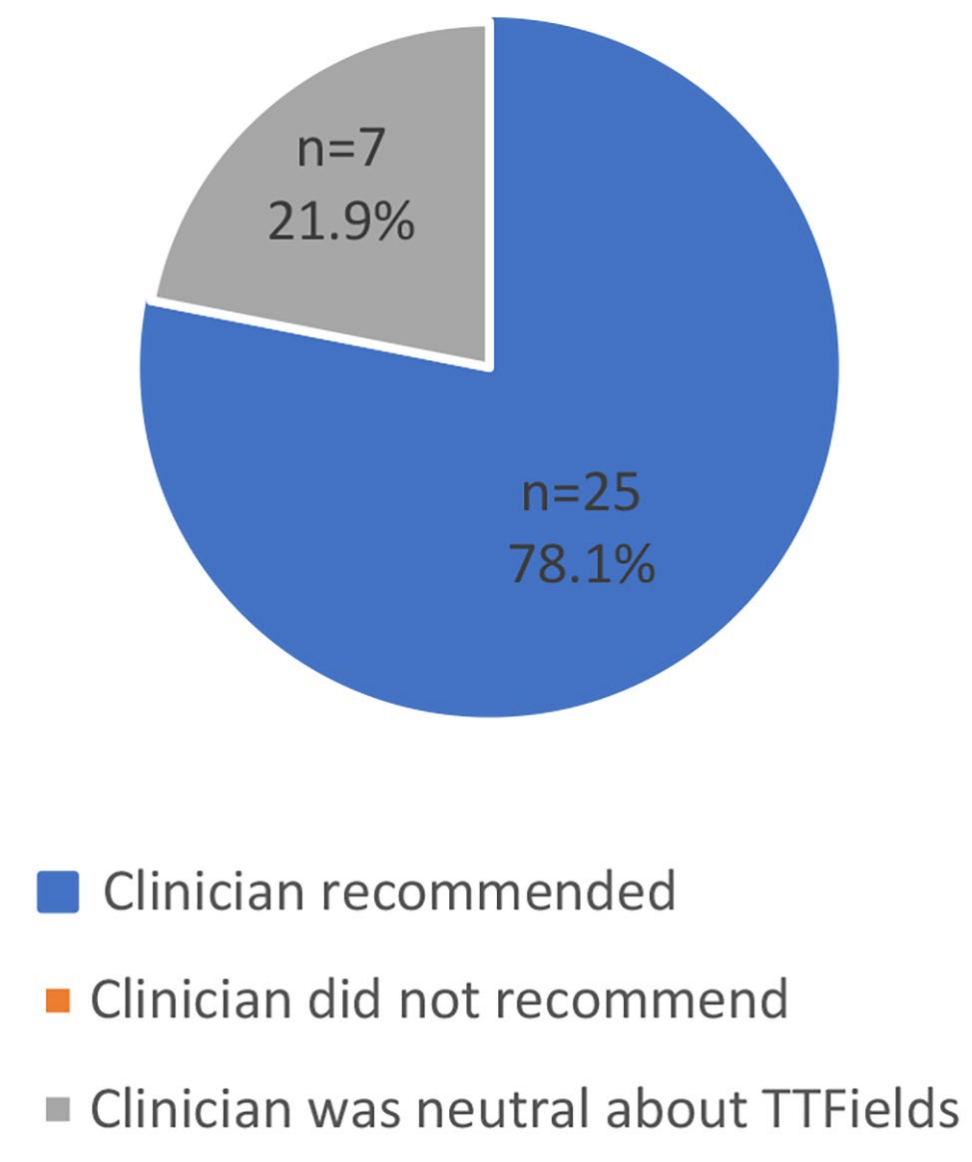
Clinician’s Recommendations for TTFields: Patients who Chose TTFields (*N* = 32)

Among participants who chose TTFields, the majority reported that their clinician recommended TTFields (*n* = 25, 78.1%) (Fig. 1). The remaining seven (58.3%) participants who chose TTFields said their clinician was neutral towards TTFields. Participants described their physicians’ neutrality as not “pushing” or wanting to “force” a decision on them. According to one participant, “[My doctor] didn’t want to push it, he did say, ‘It’s just another tool in the toolbox,’ and I’m one of these guys who wants to use all the tools.”

**Fig. 2:**
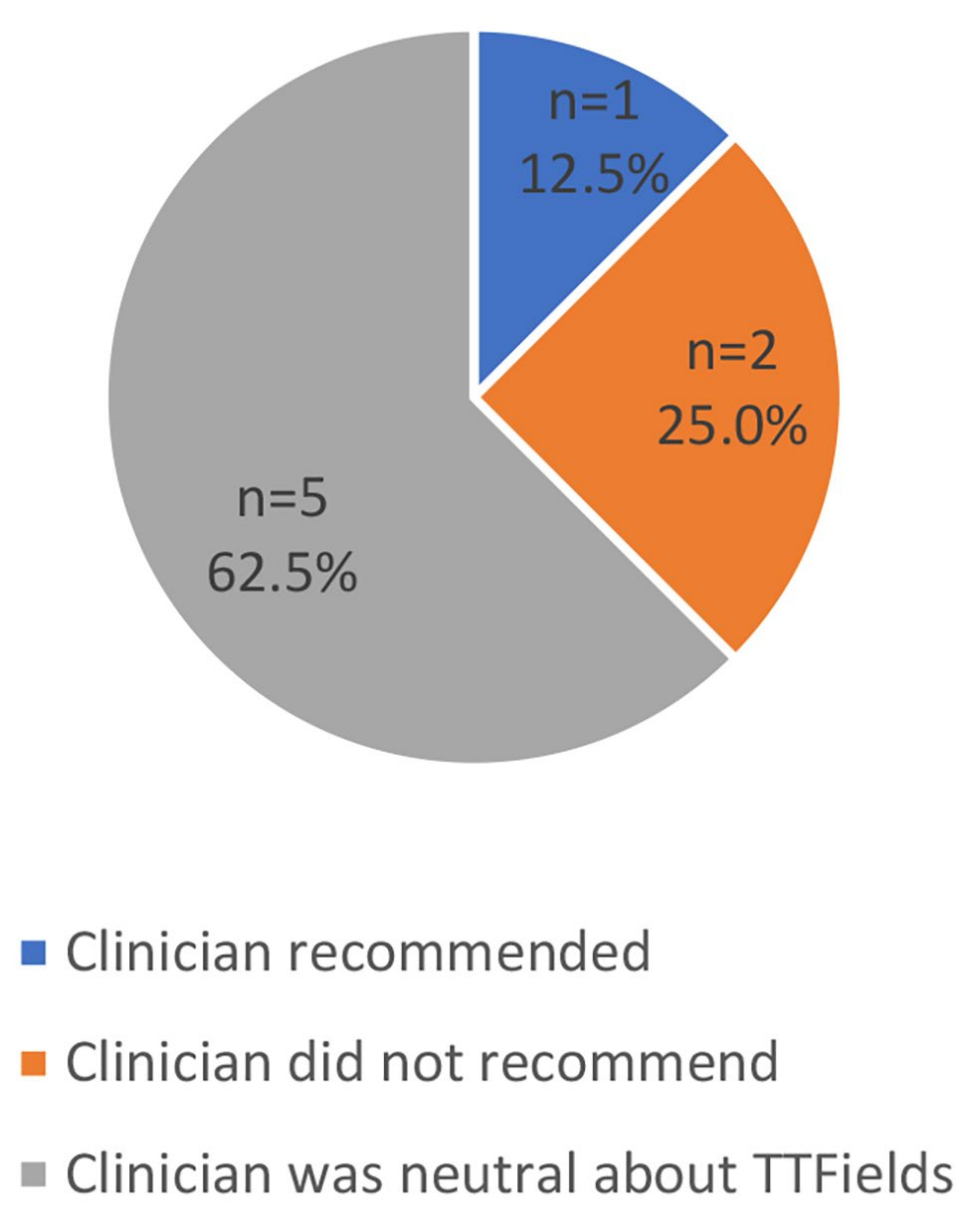
Clinician’s Recommendations for TTFields: Patients who did not Choose TTFields (*N* = 8)

For those who did not choose TTFields, 5 (62.5%) reported that their clinician was neutral regarding the patient’s decision, 2 (25.0%) reported that their clinician advised against TTFields, and 1 (12.5%) reported that their clinician recommended TTFields (Fig. 2). While the 2 participants whose clinician advised against TTFields did not report their doctor’s opinion as the most influential factor in their decision, one of them stated, “I will use the Optune (TTFields) device if the doctor recommends it.”

One participant who did not utilize TTFields described how their clinician presented the information about TTFields:

He had also said it’s the type of thing where you have to really commit to it long-term. And, he also said he likes for his patients to really feel committed to it if they want to do it, just because it’s going to be more effective if you really buy into it. And if I didn’t feel like I was there, then that was okay.

Only one participant went against their doctor’s recommendation by deciding not to undergo TTFields. This participant did not want to wear the TTFields device while working, shave their head, or wear the backpack to carry the device. The most influential factor for this participant, however, was their belief that their cancer would not come back after initial treatment. At the time of the interview, this participant was re-considering TTFields and awaiting the doctor’s recommendation based on pending test results.

### Clinician results

Clinician participants (*N* = 9) were predominantly male (*n* = 6, 66.7%) and included five neuro-oncologists, three advanced practitioners, and a physician assistant. The clinicians had been treating glioblastoma patients for an average of 14 years (range 1–30 years) and saw an average of 38 glioblastoma patients per month (range 17–60 patients). In a typical month, they treated an average of 11 glioblastoma patients with TTFields (range 1–25 patients).

### Views of TTFields: benefits and caveats

When asked if they considered TTFields to be a good treatment option for glioblastoma patients, every clinician (*N* = 9, 100%) said yes, although their enthusiasm for TTFields varied. Clinicians noted that it was a good option because it has been shown to have an impact, there are few treatment options available, and it is less toxic than other treatment options. Below are example comments from clinicians regarding the benefits of TTFields:

No question (it is a good treatment option). All of the treatment options have somewhat modest efficacy but it’s better than nothing. I will tell them when I start a treatment, when all we had was radiation, I had 90% of my patients dead within less than two years. Now, I have close to 50% of patients doing well at two years.

I think that’s pretty straightforward from my perspective. It’s a good treatment because it’s a treatment that’s been shown to improve progression-free survival and improve landmark survival…So, you put those points together and it seems as if it has a real impact.

I try not to say in so many words that we don’t have a lot of options for them with this disease and that this is one that we know works for whatever true benefit it provides for each patient, we know that it provides a benefit, and that of the options we have, this is the least-toxic, the one that will be the least likely to cause them any ill effects, will mold into their lifestyles the easiest, and that it does work.

However, some clinicians noted caveats to their endorsement of TTFields. For example, some noted that TTFields is not feasible for certain patients, such as the elderly or patients with poor performance status. This clinician noted the best candidates are patients who have a caregiver to help them with the device or are very functional and motivated to manage the device themselves. Likewise, another clinician emphasized the need for patient motivation to use TTFields:

This (TTFields) is kind of part of you all the time, so I think people just have to kind of accept that. I think the website does a good job of showing people living their normal life with the device, so if people feel like they can be like that, then I think it’s a great treatment, but they have to be willing… and buy into shaving their head, changing the arrays, having the device on 18-plus hours a day, taking it with them when they leave the house, being connected to the plug when they’re at home.

Several clinicians described a neutral approach to advising patients about TTFields. These clinicians said that evaluating whether the burden of TTFields is worth the benefit is the patient’s decision.

I typically frame it in a way where if the patient feels that the burden outweighs the benefit, then they’re the ones in the driver’s seat and they can say, I don’t want to do it because of their perception that this has too much of a negative impact on their quality of life.

One oncologist noted that patients’ views of TTFields are influenced by how clinicians present TTFields:

I think none of us have hesitation presenting to somebody whom we meet who we suspect has a brain tumor that, “You need surgery. There’s no way around this.” Right? And so, we tell them that and we’re comfortable. We have no problem telling them, “You need radiation therapy, or we need to give you this chemotherapy.” But I think culturally, within the neuro-oncology community, there is some hesitancy to say, “Oh, you also need TTFields.” It’s strange to me that that’s the case, because all these other things that are much bigger-gun therapeutics with higher risk profiles, we have no problem recommending.

When asked why clinicians are hesitant to endorse TTFields, this oncologist noted two reasons. First, he noted that changing behavior that is ingrained in one’s work culture is difficult. Second, TTFields is foreign to the oncology world:

Clinician: I think the other aspect that makes tumor treating fields a little bit strange and odd is…it’s coming from the electrical engineering world. And almost none of us have a background in that….

Interviewer: Okay. Do you think physicians are hesitant to use TTFields because they doubt the evidence behind it, or just because it is different?

Clinician: I think some people doubt the evidence and I think it’s easier to doubt the evidence when something is different. When you have an outlier idea, the body of proof that you need to and should have to present becomes much higher. So, if you’re presenting a concept that’s not that far removed from everything else that is rapidly accepted as dogma, the burden of proof doesn’t have to be huge… I think there are critics that come at it from the neuro-oncology community that it’s a treatment that didn’t have a sham control, which one could argue we had no problem accepting the lack of blinded study design for temozolomide, which everyone is comfortable administering. So, we have a precedent, but I think people sometimes forget that.

When were asked if they would recommend TTFields for a family member or use it themselves if they had glioblastoma, four of the nine (44%) clinicians said yes, citing reasons such as the minimal risks and the desire to do everything possible to extend their life. Three clinicians (33%) said maybe, noting that it would depend upon whether they would want to go to hospice or obtain treatment, upon the condition of themself or their loved one, and that they probably would not want to be attached to the device but if in that situation they may feel differently. Two clinicians (22%) said no, they would not use TTFields themselves or recommend it for a family member. Of the two who said no, one explained that they would not want to extend life in a compromised state; three months of survival without TTFields vs. 15 months of survival on TTFields was not worth the burden of the device. Similarly, the other physician noted that the added survival does not outweigh the negative quality of life impacts of TTFields.

### Discussing survival benefits of TTFields

All clinicians noted that TTFields has a proven benefit for patient lifespan. When asked how they describe the lifespan benefits to patients, clinicians described TTFields benefits in general terms, as evidenced in the quotes below:

(I tell patients) it has sort of become a standard of care for patients after radiation. There’s data that says it improves overall survival and most times it is tolerated pretty well.

I don’t (talk to them about benefits to their lifespan), except to say generally that it can add—that really the advantage is that it can buy them some time that is of quality. The problem is that if you talk about time, the time itself isn’t super-significant, in my opinion. And everyone is different. I don’t know what someone’s progression might be with or without Optune (TTFields)… I do tell them that it will add time to their lives, theoretically…but I can’t talk about specific times.

One clinician noted that he tells patients that TTFields can increase two-year survival. However, he felt that discussing months of life extension was not appropriate:

I usually don’t quote months because I think at that moment (the initial meeting) they’re probably too fragile…I rarely quote months at the initial meeting. So, I keep things broader. I don’t say, ‘Well data supports that using this, it improves survival by four months or something.’ I don’t say that.

Another clinician also felt that it would be detrimental to patients to tell them the number of months of extended life provided by TTFields:

It’s tough to do without deflating the patient. And the goal is to infuse the patient with a reasonable hope and not overinflate their expectations. So, keep it reasonable, but at the same time give them things that I feel hopeful about.

Clinicians noted that they typically provided specific data on life extension only if asked. As one clinician noted, “If the patient asks for specific data around the treatment, I’m happy to give that to them. But I tend to avoid talking about statistics.” According to another clinician, “I would say it’s the rare patient who wants a lot more detail than– they usually don’t want to get super-scientific about it.”

## Discussion

This is the first study of views of TTFields among glioblastoma patients and clinicians. Our findings indicate that TTFields is burdensome for patients who use the device, particularly due to changing the arrays and carrying the device. Because there is no cure for glioblastomas, treatment decisions involve prioritizing length of life (LOL) or quality of life (QOL). However, our participants who chose TTFields reported a willingness to accept the burden of TTFields because of its proven efficacy. These results are consistent with the phase three clinical trials of TTFields in which patients receiving TTFields did not report worse social role, social functioning, or physical functioning despite the physical burden of carrying the device and the social impact of the visibility of the device [17]. Thus, for patients motivated to use this device, the quality of life burden may not be excessive [18].

Patients who declined TTFields stated that its QOL impacts, such as shaving one’s head or the visibility of TTFields, outweighed the potential LOL benefits. Interestingly, many participants who declined TTFields reported not knowing its impact on survival. It may be that for these patients, no extension of life would outweigh the burden of TTFields. These findings are consistent with observations made by other glioblastoma research: “It appears that, for some, the inconvenience and/or possible stigma associated with the device may be easily mitigated by the potential survival benefit; however, for others, a survival benefit without the promise of a cure may not justify the need for continuous use of the device.” [12] (p.859).

When presenting TTFields to their glioblastoma patients, clinicians in this sample typically described TTFields’s lifespan advantages in general terms rather than by providing specific prognostic information. Some clinicians noted a desire to balance the fragility of patients diagnosed with a terminal illness with the need to provide information. These findings are consistent with prior work on decision making in advanced cancer wherein clinicians are hesitant to discuss survival and treatment outcomes, [19–21] physicians make implicit judgements about patients’ desired level of information, [22] and the central characteristics of shared decision making are often absent [23]. Although advanced cancer patients, including glioblastoma patients, often report satisfaction with treatment decision making [23], most patients with incurable cancer want information on life expectancy, including typical, best, and worst case scenarios [24–27]. Moreover, awareness of cancer prognosis is linked to improved patient quality of life, goal-concordant care, and lower healthcare costs [20, 28, 29]. Thus, glioblastoma patients may benefit from tools designed to assist them in asking questions about their prognosis [30].

Patients’ preferred decision-making roles may impact the decision to use TTFields. Patients who declined TTFields were more likely to report a preference for independent decision-making. This preliminary finding should be confirmed in future work with a larger sample of patients. Additionally, these data suggest clinicians could utilize patient decision making preferences to frame the discussion when presenting the TTFields option.

Patients’ TTFields decisions tended to align with their perception of what the physician recommended for them. Clinicians in our sample were generally supportive of using TTFields for glioblastoma patients. The level of support for TTFields among clinicians in this sample is unusual; [12] future work should consider clinician support of TTFields in other institutions and with larger samples. Our clinician sample noted that TTFields was an appropriate treatment option for glioblastoma because it was shown to be efficacious, there are few treatment options available for glioblastoma, and it is less toxic than other treatment options. While some clinicians enthusiastically endorsed TTFields for their eligible patients, others presented it as an option but noted that they want patients to consider whether the burden of TTFields is worth the benefits of the treatment and emphasized that patients needed to be motivated to commit to using the device.

Our sample consisted primarily of patients who chose to use TTFields. The large number of TTFields users in our sample is due, in part, to the fact that patients interested in trying all forms of treatment often seek out clinicians at the Malnati Brain Tumor Institute. Additionally, patients who declined TTFields may have been in poorer health and deemed ineligible for this study. Additional work is needed to confirm preliminary findings among a sample of patients who decline TTFields. Finally, future work can build upon our findings to develop decision tools for patients and clinicians considering TTFields. These tools should incorporate patients’ desired decision-making role and assess patients’ openness to receiving specific information about survival benefits.

## Conclusion

Qualitative research within healthcare aims to uncover meaning, listen to the perspectives of key stakeholders, and build theories [31]. Without qualitative exploration, subsequent quantitative work may overlook or misrepresent key factors for a particular health problem. We sought to identify factors in the social environment or in patient beliefs that shape TTFields decisions for adult glioblastoma patients.

The decision to use TTFields is a highly personal one for glioblastoma patients. Our findings, which come from the first study of views of TTFields among glioblastoma patients and clinicians, indicate that for some patients who refuse the device, the extension of life gained from using TTFields cannot outweigh the burden of the device. However, the quality-of-life burden of the device may not be excessive for patients who are motivated to use it. Increasing provider support of TTFields may lead to increased use of the device as patients’ TTFields decisions tended to align with their perception of what the physician recommended. Future work should consider the extent to which glioblastoma patients desire more specific prognostic information.

## Data Availability

The datasets used and/or analysed during the current study are available from the corresponding author on reasonable request.

## Abbreviations

TTFields: Tumor Treating Fields
TMZ: Temozolomide
NCCN: National Comprehensive Cancer Network
LOL: Length of life
QOL: Quality of life
